# One year after the COVID-19: What have we learnt, what shall we do next?

**DOI:** 10.1192/j.eurpsy.2021.9

**Published:** 2021-02-15

**Authors:** Philip Gorwood, Andrea Fiorillo

**Affiliations:** 1 INSERM, U1266 (Institute of Psychiatry and Neuroscience of Paris), Université de Paris, Paris, France; 2 CMME, GHU Paris Psychiatrie et Neurosciences, Hôpital Sainte-Anne, Paris, France; 3 Department of Psychiatry, University of Campania L. Vanvitelli, Naples, Italy

We are still within the acute phase of the COVID-19 pandemic, so getting lessons of how we coped with it during its first year of life is of interest. The European Psychiatric Association has been trying to help patients, caregivers, and policy makers to concentrate their efforts on those most at risk, relying on consistent information, taking into account many underestimated aspects and making proposals to facilitate adequate strategies. The *European Psychiatry* journal, being the official platform of our association, presents a collection of articles devoted to COVID-19 in line with these priorities.

The first important aspect of this collection is how COVID-19 addresses the topic of psychiatric symptoms in the general population, and how such increase mainly concerns those with past or present mental disorders. Care providers are also at risk of psychiatric symptoms according to a large study in China, with one out of four of respondents experiencing high levels of anxiety or/and depressive symptoms [1]. For the global population, one in three to six people appear adversely affected by depression, anxiety, insomnia, or suicidal ideas, the strongest predictor of these disorders being a history of mental health problems [2]. Another interesting study carried out in Italy [3] showed that with longer time being in a lockdown situation, there is a very significant increase of the level of depressive, anxiety, and stress symptoms. Once again, subjects with pre-existing mental health problems were at even higher risk. Moser et al. [4] analyzed these aspects using the concept of years of life lost. They show that the “average” person would lose 70 days of life due to psychosocial consequences of COVID-19 mitigation measures, and that this loss would be entirely borne by 2% of the population, who will suffer an average of 10 years lost.

The road to hell is paved with good intentions, so when we try to acutely adapt our system to a worldwide crisis, we have to be careful not worsening the situation. An example is the use of the term “social distancing,” widely used to reduce the spread of the virus. But this term evokes negative feelings of being ignored, unwelcome, and even excluded from society, leading Wasserman et al. [5] to propose using the term “physical distancing.” Taking into account the fact that some patients might have difficulties of wearing a face mask is also having potential side effects. Some Governments proposed, for example, that those “*presenting behavioral alterations”* (Spain) or *“because of a mental illness”* (UK) could constitute exemptions to the rule [6]. This well-intended proposal might be a bad idea, as there is no evidence that face masks affect mental health in a negative way, and such exemption may carry an increased risk of COVID-19 for all patients with mental disorders who are at higher risk of infection, and with higher rates of hospitalization and death compared to the general population [6]. In order to facilitate treatments in isolated wards because of the COVID-19, a discontinuation of judges’ visits to patients was observed in many countries, including Germany and France. Thome et al. [7] raised the point that this is also a damageful reduction of rights of patients being hospitalized that should not be accepted, the need for a “pandethics” being put to the forth.

Three articles of this collection listed advices and recommendations to protect and defend the rights of patients with severe mental disorders in the circumstances of a pandemic [7], to organize basic principles of mental healthcare during the COVID-19 pandemic [8], and to help the general population to reduce stress and cope with related aspects such as confinement and lockdown [9].

An efficient fight against the virus means isolating those infected, increasing the protection of all citizens, but even more those at higher risk. This means that subjects with serious mental disorders should be considered as among those needing protection, support, and vaccine with the highest level of priority.
